# Antimony Potassium Tartrate Stabilizes Wide-Bandgap Perovskites for Inverted 4-T All-Perovskite Tandem Solar Cells with Efficiencies over 26%

**DOI:** 10.1007/s40820-023-01078-6

**Published:** 2023-04-14

**Authors:** Xuzhi Hu, Jiashuai Li, Chen Wang, Hongsen Cui, Yongjie Liu, Shun Zhou, Hongling Guan, Weijun Ke, Chen Tao, Guojia Fang

**Affiliations:** Key Laboratory of Artificial Micro/Nano Structures of Ministry of Education, School of Physics and Technology, Wuhan University, Wuhan, 430072 People’s Republic of China

**Keywords:** Perovskite solar cell, Tandem, Wide bandgap, Multifunctional additive

## Abstract

Wide-bandgap (WBG) perovskites have been attracting much attention because of their immense potential as a front light-absorber for tandem solar cells. However, WBG perovskite solar cells (PSCs) generally exhibit undesired large open-circuit voltage (*V*_OC_) loss due to light-induced phase segregation and severe non-radiative recombination loss. Herein, antimony potassium tartrate (APTA) is added to perovskite precursor as a multifunctional additive that not only coordinates with unbonded lead but also inhibits the migration of halogen in perovskite, which results in suppressed non-radiative recombination, inhibited phase segregation and better band energy alignment. Therefore, a APTA auxiliary WBG PSC with a champion photoelectric conversion efficiency of 20.35% and less hysteresis is presented. They maintain 80% of their initial efficiencies under 100 mW cm^−2^ white light illumination in nitrogen after 1,000 h. Furthermore, by combining a semi-transparent WBG perovskite front cell with a narrow-bandgap tin–lead PSC, a perovskite/perovskite four-terminal tandem solar cell with an efficiency over 26% is achieved. Our work provides a feasible approach for the fabrication of efficient tandem solar cells.

**Supplementary Information:**

The online version contains supplementary material available at 10.1007/s40820-023-01078-6.

## Highlights

- A versatile material-antimony potassium tartrate is added to wide-bandgap perovskite cells for the first time.
- Inverted wide-bandgap perovskite solar cells with a structure of ITO/MeO-2PACz/perovskite/C_60_/ALD-SnO_2_/Cu yield a champion power conversion efficiency of 20.35%.
- A perovskite/perovskite four-terminal tandem solar cell with efficiency of 26.3% is achieved.

**Supplementary Information:**

The online version contains supplementary material available at 10.1007/s40820-023-01078-6.

## Introduction

In the past decade, certified power conversion efficiency (PCE) of single-junction organic–inorganic hybrid perovskite solar cells (PSCs) has been skyrocketing over 25% [1–5]. To further improve the efficiency and stability of PSCs, strategies such as interface engineering, bandgap regulation, and structural design are widely proposed [6–10]. Tandem solar cells, consisting of a wide-bandgap (WBG) perovskite front cell and a bottom cell with a narrow-bandgap light absorber such as narrow-bandgap perovskites [11–14], silicon [15–18], Cu (In, Ga) Se_2_ [19, 20], and organic semiconductors [21, 22], have been demonstrated to be an effective strategy to break the Shockley–Queisser efficiency limit of single-junction cells.

Efficient and stable WBG PSCs are prerequisite and decisive to obtain efficient perovskite-based tandem solar cells. However, the development of WBG perovskite films and related photovoltaics processed from solution method usually suffers several limitations. One is that they have high defect densities at grain boundary and surface, which results in large open-circuit voltage (*V*_OC_) deficits (*E*_g_/e–*V*_OC_), ion migrations and low phase stability [23–27]. The other is that obvious hysteresis is exist resulting from unfavorable carrier transport due to interface band energy-level mismatches. In recent years, much effort has been devoted to reduce the defect density of WBG perovskite films, including surface, composition or additive engineering [20, 23–25, 28, 29]. For the surface engineering, various techniques including 2D perovskite top layer passivation have been developed to passivate the surface or even the body defects. For example, Chen et al*.* employed 2-thiopheneethylammonium chloride on top of the perovskite layer to form a 2D perovskite passivation layer in inverted WBG PSCs, improving the optoelectronic properties of the perovskite absorber and passivating both electron and hole traps and hence achieving a *V*_OC_ of 1.19 V in WBG PSCs [24]. For the composition engineering, for example, Wen et al*.* obtained WBG perovskites with lowered bromide contents by composition engineering of alloying dimethylammonium and chloride into mixed-cation and mixed-halide perovskites. Consequently, a high PCE of 17.7% and an increased *V*_OC_ of 1.26 V were obtained in WBG single-junction cells [11].

The introduction of additives in perovskite precursor solutions which demonstrated as one or bifunctional improvement has been proven to be an effective strategy for obtaining high-quality perovskite films and high-performance devices [30–32]. For example, Yu et al*.* reported that 4-fluoro-phenylethylammonium iodide as a bifunction additive promoted perovskite crystals to grow along with the (100) orientation and comprehensively passivated WBG perovskites, achieving a *V*_OC_ of 1.30 V in WBG PSCs [33]. Potassium halides have been introduced to WBG perovskites to passivate defects, suppress ion migrations [32, 34, 35]. Thus, phase segregation and hysteresis were greatly suppressed [23, 36].

The reported additives include inorganic materials, small organic molecules, polymers, organic halides and acids. In addition, a large number of organic molecules containing sulfhydryl, carboxyl and amino functional groups have been reported to passivate defects by interacting with PbI_2_ in perovskite precursors. Wang et al. recently added tartaric acid (TA) into the perovskite precursor solution to adjust the crystal growth, resulting in high-quality perovskite films with enhanced preferred orientations [37]. The TA additive improved the energy-level arrangement of PSCs, thus effectively accelerating the carrier extraction together with inhibiting the non-radiative recombination at perovskite/carrier transporting layer interface. Thanks to the carboxyl group (–COOH) regulating crystal growth dynamics and suppressing Sn oxidation, TA had also been successfully added to perovskite precursor solutions recently to stabilize the crystal structure of Sn–Pb perovskites [38]. However, the intrinsic mechanism of TA and its derivatives interacting with WBG perovskites and their applications in all-perovskite tandem solar cells have not been investigated yet. In the meantime, a multifunctional additive is highly desired to synergistically passivate the body, surface defects and modulate the interfacial energy levels of perovskite solar cells.

In this work, antimony potassium tartrate (APTA) is added into WBG perovskite precursor as a multifunctional additive that not only suppresses non-radiative recombination but also inhibits phase segregation and modulate perovskite energy levels, which results in enhancement of carrier lifetime and stability of perovskite, better band energy alignment and therefore suppressed hysteresis. Consequently, the APTA auxiliary WBG PSC delivers a maximum PCE of 20.35% with negligible hysteresis. Under 100 mW cm^−2^ white light illumination in nitrogen after 1,000 h, they maintain 80% of their initial efficiency. Moreover, by combining a semi-transparent WBG perovskite front cell with a narrow-bandgap tin–lead PSC, a perovskite/perovskite four-terminal (4-T) tandem solar cell with an efficiency over 26% is achieved. Our work demonstrates a feasible scheme to accomplish efficient tandem solar cells.

## Experimental Section

### Materials

Lead iodide (PbI_2_, 99.99%) and MeO-2PACz were purchased from TCI. Lead bromide (PbBr_2_, 99.999%) and cesium iodide (CsI, 99.999%) were purchased from Alfa Aesar. Formamidinium iodide (FAI, 99.99%) was purchased from Ying Kou You Xuan Trade Co. Ltd. Potassium Antimony Tartrate was purchased from Sinopharm Chemical Reagent Co., Ltd. C60 was purchased from Xi’an Polymer Light Technology Corp. DMF (99.99%), DMSO (99.99%), chlorobenzene (CB, 99.9%), and methanol (99.9%) were purchased from Sigma-Aldrich. Diethyl ether (98%) was purchased from Yonghua Chemical Co. Ltd. All chemicals and reagents are used without any further purification.

### Device Fabrication

#### Fabrication of WBG Perovskite Solar Cells

The patterned ITO substrates were ultrasonically cleaned in detergent, deionized water, acetone, and ethanol for 15 min, respectively. The samples were blown dry with nitrogen (N_2_), followed by being treated with ultraviolet ozone for 15 min. The samples were then transferred to an N_2_-filled glove box. Next, MeO-2PACz solution (0.3 mg mL^−1^ in ethanol) was spin-coated onto ITO at 3,000 rpm for 30 s following by annealing at 100 °C for 10 min. The WBG perovskite precursor was prepared by mixing CsI, FAI, PbI_2_, PbBr_2_ in DMF: DMSO (3:1/V:V) with a concentration of 1.4 M. For the target samples, antimony potassium tartrate with various concentrations was added to 1 mL precursor. The perovskite precursor solution was then deposited onto MeO-2PACz layer at the first step with 500 rpm for 2 s and the second step with 4,000 rpm for 60 s. Before the end of the spin coating, 500 µL of diethyl ether was added dropwise at 35 s after the start of the second process. The samples were then annealed at 100 °C for 5 min. All of the perovskite films were prepared under the same condition. After cooling down, C_60_ (20 nm) was deposited by thermal evaporation under a vacuum pressure of 9.0 × 10^−5^ Pa.

For the ALD process, a buffer layer of 20 nm thick SnO_2_ was deposited at 90 ºC, using tetrakis(dimethylamino) tin (TDMA-Sn, Strem chemicals, 99.99%) and ultrapure water as the precursors. The 130 cycles ALD of SnO_2_ consisted of the processing sequence: 15 ms TDMA-Sn pulse, 15 s purge, 10 ms H_2_O pulse, and 15 s purge under 20 sccm N_2_ as the carrier gas.

For the opaque devices, Cu (100 nm) was deposited onto ALD-SnO_2_ by thermal evaporation through a shadow mask with an active area of 0.1188 cm^2^. For the semi-transparent devices, 100-nm Cu electrode was replaced by 100-nm ITO film by magnetron sputtering at 100 W.

#### Fabrication of NBG Perovskite Solar Cells

The ITO substrate was cleaned in the same way as mentioned above. The filtered PEDOT: PSS solution was spin-coated on the ITO substrates at 4,000 rpm for 30 s and then annealed at 140 °C for 20 min. Subsequently the filtered FA_0.7_MA_0.3_Pb_0.5_Sn_0.5_I_3_ precursors were deposited on the PEDOT: PSS-coated substrates by a two-step spin-coating process, including 1,000 rpm for 10 s and then 4,000 rpm for 40 s. 400 µL of CB was dropped onto the spinning substrates during the second spin-coating step at 20 s before the end of the procedure. The substrates were then immediately annealed on a hot plate at 100 °C for 10 min, followed by annealing at 65 °C for 12 min in a glove box. Finally, C_60_ (20 nm)/BCP (7 nm)/Cu (80 nm) were sequentially deposited on the substrates by thermal evaporation to complete the fabrication.

### Characterizations

#### Film Characterizations

The absorption spectra of perovskite films and precursor solutions were performed using a spectrophotometer (UV–vis, miniUV-1208 model, Shimadzu). The steady-state photoluminescence (PL) and time-resolved photoluminescence (TRPL) spectra were measured using a DeltaFlex fluorescence spectrometer (HORIBA) with a semiconductor intensity to 1 sun equivalent intensity at 481 nm. The crystal structure of the perovskite polycrystalline films was analyzed by an X-ray diffractometer (D8 Advance, Bruker). The top-view and cross-sectional images of the perovskite films and devices were observed with a field emission scanning electron microscope (Zeiss SIGMA). XPS and UPS spectra were measured using an XPS/UPS system (Thermo Scientific, Escalab 250Xi).

#### Device Characterizations

*J*–*V* characterizations and steady-state power outputs (SPO) of the PSCs were performed under air mass 1.5 global (AM 1.5 G) conditions (Enli Technology Co. Ltd) in an nitrogen atmosphere. The *J–V* measurements were performed with a scan rate of 20 mV s^−1^ ranging from 1.2 to −0.2 V and then reversed again from −0.1 to 1.2 V. Incident photoelectric conversion efficiency (IPCE) was recorded with a QE/IPCE system (Enli Technology Co. Ltd). For 4-T tandem solar cell measurements, we firstly measured PCEs of top semi-transparent WBG perovskite solar cells and then those of the bottom narrow-bandgap ones, which were optically filtered by the top semi-transparent WBG ones. The PCEs of 4-T all-perovskite solar cells were the sum of the efficiencies of top cells and bottom ones. The SCLC of the single carrier devices was measured using a CHI660E electrochemical workstation (Shanghai Chenhua Instruments, Inc). The Mott–Schottky curve with capacitance–voltage measurements was carried out by a CHI760E electrochemical workstation (Shanghai Chenhua Instruments, Inc) at 1,000 Hz with the bias voltages ranging from 1.4 to 0 V and an AC voltage of 0.02 V was used to test the corresponding capacitance at shifty bias voltage. Admittance spectroscopy measurements were performed with the CHI760E electrochemical workstation at different temperatures ranging from 250 to 320 K in the dark at a low vacuum (≈10 Pa) with a small AC disturbed voltage of 20 mV. The frequency scope from 1 to 100 kHz and a DC bias voltage of 0 V was kept during the measurements.

## Results and Discussion

### Crystal Quality and Structure Analysis of Perovskite Films

**Fig. 1:**
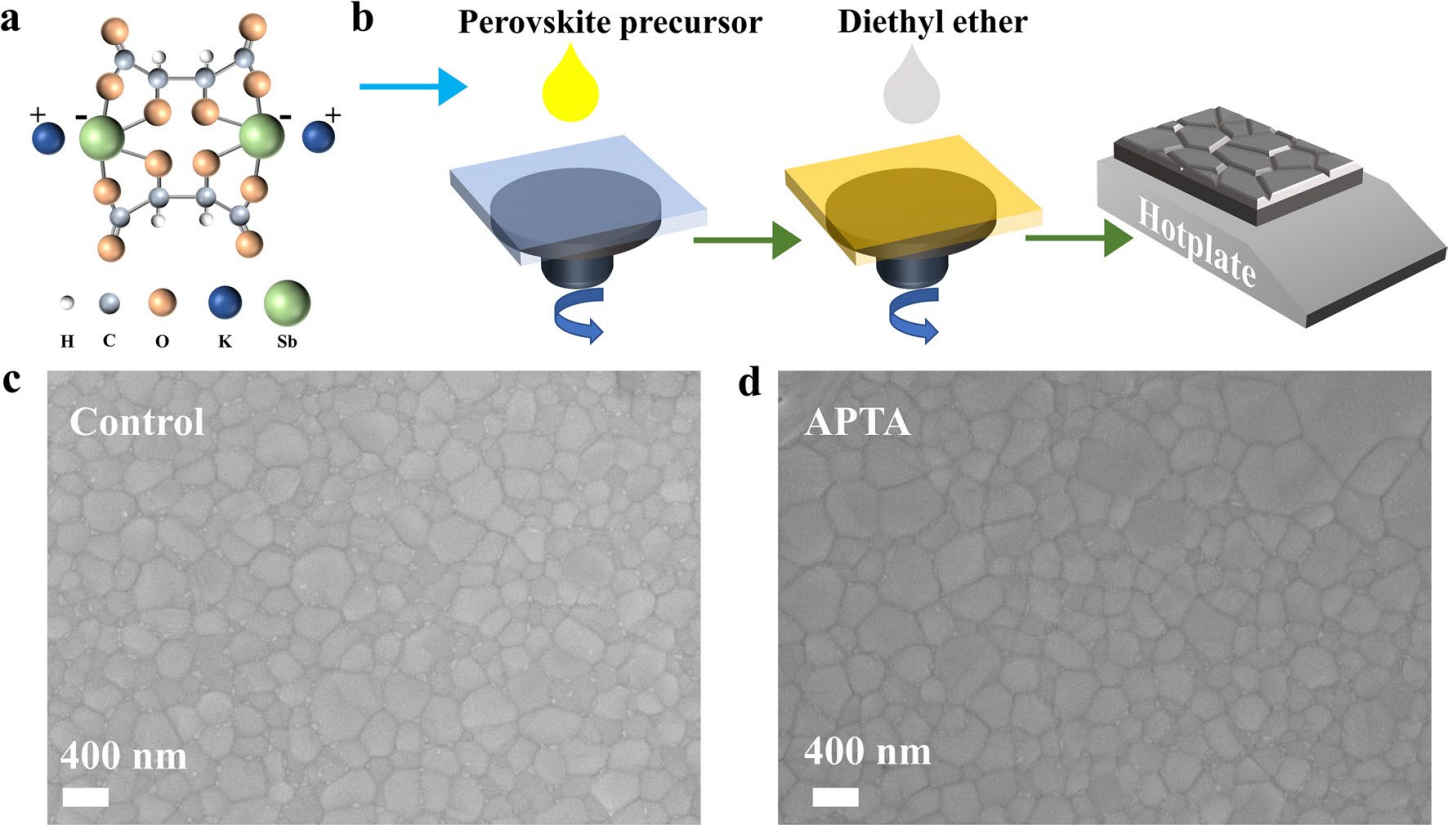
**a** Molecular structure of APTA. **b** Schematic illustration of the fabrication process for the WBG perovskite films. Top-view SEM images of WBG perovskite films **c** without and **d** with APTA

Figure 1a shows the APTA’s molecular structure and Fig. 1b schematically illustrates the so-called one-step deposition fabrication process for the perovskite films, i.e., anti-solvent treatment and then thermal annealing at 100 ºC. In this work, FA_0.75_Cs_0.25_Pb(I_0.8_Br_0.2_)_3_ perovskite with a high Br content was used as WBG perovskite absorber. In order to examine the effect of APTA additives on perovskite film morphology, top-view scanning electron microscopy (SEM) images of perovskite films were taken and displayed in Fig. 1c, d. Both the control and target perovskite films are flat and uniform without pinholes. However, the perovskite film with APTA showed a larger grain size, which suggests APTA promotes the crystallization of perovskites. Subsequently, we characterized the corresponding atomic force microscopy (AFM) images and analyzed the impact of APTA on perovskite film morphology. In Fig. S1, the root-mean-square (RMS) roughness of the perovskite film with APTA is 20.1 nm, which is less than that of the control (25.5 nm), further indicating that the addition of APTA helps to form a flatter perovskite film.

**Fig. 2:**
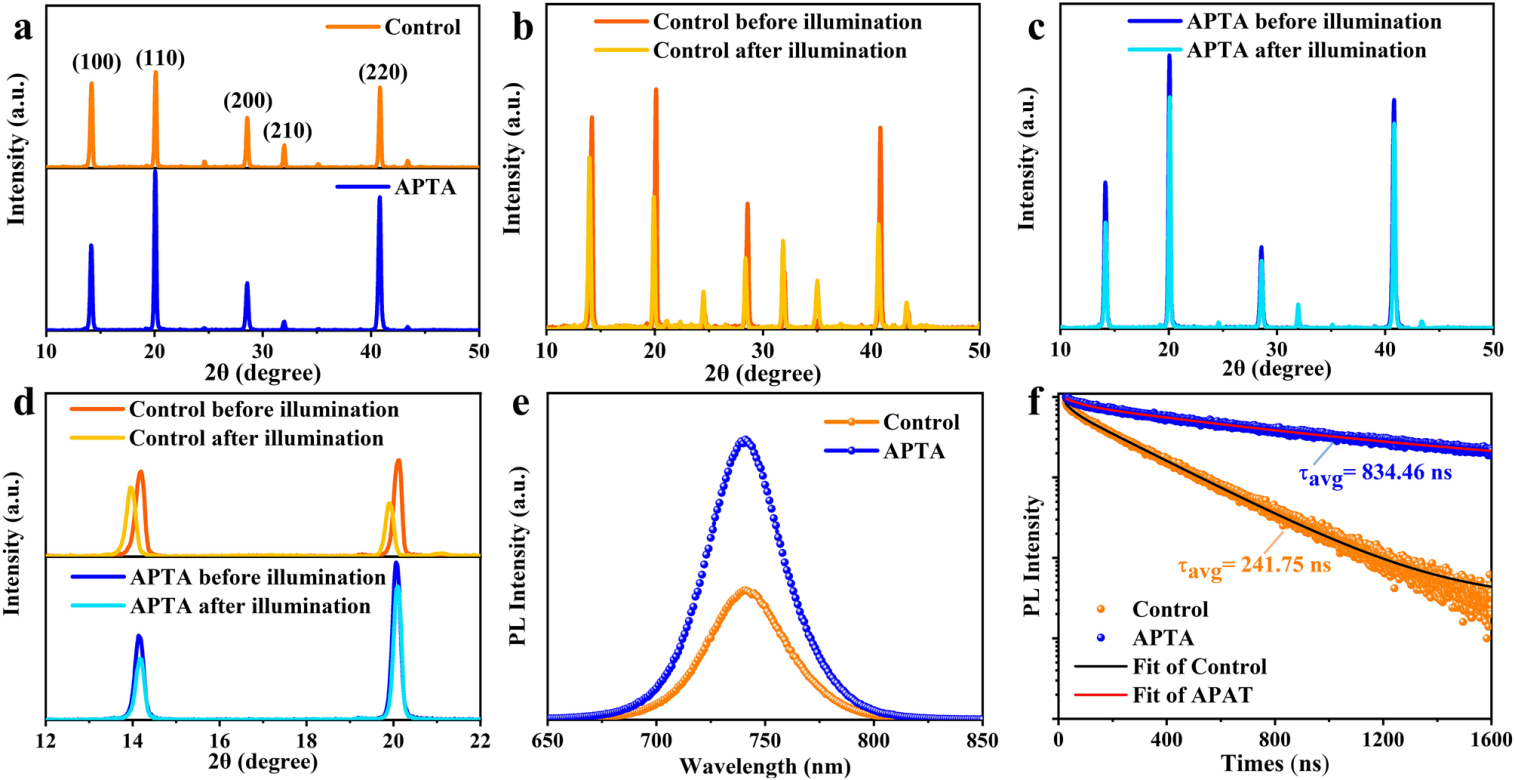
**a** XRD patterns of the WBG perovskite films without and with APTA. The evolution of XRD patterns of the WBG perovskite films **b** without and **c** with APTA upon continuous light illumination. **d** Enlarged XRD patterns of the WBG perovskites w/o and with APTA before and after continuous light illumination. **e** Steady-state photoluminescence spectra (PL) of WBG perovskites with and without APTA and the corresponding **f** time-resolved PL spectra

Then, the influence of APTA on the crystal structure and the crystallinity of perovskite films was investigated using X-ray diffraction (XRD). As shown in Fig. 2a, strong XRD peaks are observed at 14.17°, 20.06°, 28.57°, and 40.82°, which are ascribed to the (100), (110), (200) and (220) diffraction planes of polycrystalline perovskite films [17], respectively. The positions of these main peaks change negligibly after the incorporation of APTA, indicating that no new phase forms. However, the diffraction angles at 20.06° and 40.82° are much stronger than those of the control, which indicates that WBG perovskite film with APTA crystallizes orientationally. Subsequently, to analyze the effect of APTA additives on the light stability of perovskite films, the lattice structure evolution was investigated upon the continuous light exposure. As displayed in Fig. 2b, the main diffraction peaks of the control perovskite film shifted to lower angles after 20 min of one-sun irradiation. For instance, the diffraction peaks at 14.17° and 20.06° of the control film slightly shifted to 13.94° and 19.91°, respectively, as illustrated in the enlarged view in Fig. 2d. This change agrees well with the appearance of narrow-bandgap phase caused by halide migration [23]. By contrast, the WBG perovskite film with APTA shifts little in diffraction angles after continuous light illumination (Fig. 2c). Moreover, the magnified diffraction pattern at the bottom of Fig. 2d shows that the lattice expansion induced by light irradiation is almost absent, which means that potassium in APTA inhibits halogen migration to reduce phase segregation.

To further investigate the influence of APTA on the optical properties in WBG perovskite films, steady-state photoluminescence (PL) and the corresponding time-resolved photoluminescence (TRPL) measurements were performed in ambient air using a 481 nm continuous semiconductor laser, and the results are displayed in Fig. 2e–f. The PL peaks of both control and target WBG perovskite films locate at 742 nm, indicating that the incorporation of APTA negligibly changes the bandgap of perovskite film. Nevertheless, the PL peak intensity of the perovskite film with APTA is as twice as that of the control, implying that nonradiative recombination in perovskite film is significantly suppressed upon the addition of APTA. The evolution of PL spectra of perovskite films under continuous laser irradiation were also recorded to characterize the suppression of phase segregation. As shown in Fig. S2, the PL peak of the control film widens toward long wavelength under continuous irradiation, while that of the APTA-assisted perovskite film changes negligible. Figure 2f presents the TRPL spectra and the decay curves are well fitted by the following bi-exponential decay equation:

1 $$y = y_{0} + A_{1} e^{{ - t/\tau_{1} }} + A_{2} e^{{ - t/\tau_{2} }}$$

The main fitted data are summarized in Table S1. It is evident that the derived average lifetime (*τ*_avg_) of 834.46 ns for the perovskite film with APTA is much longer than that of the control film (241.75 ns). This suggests that the charge recombination in perovskite film is significantly suppressed due to the enhanced crystallization and/or efficient defect passivation in the presence of APTA.

### Performance of WBG Perovskite Solar Cells

**Fig. 3:**
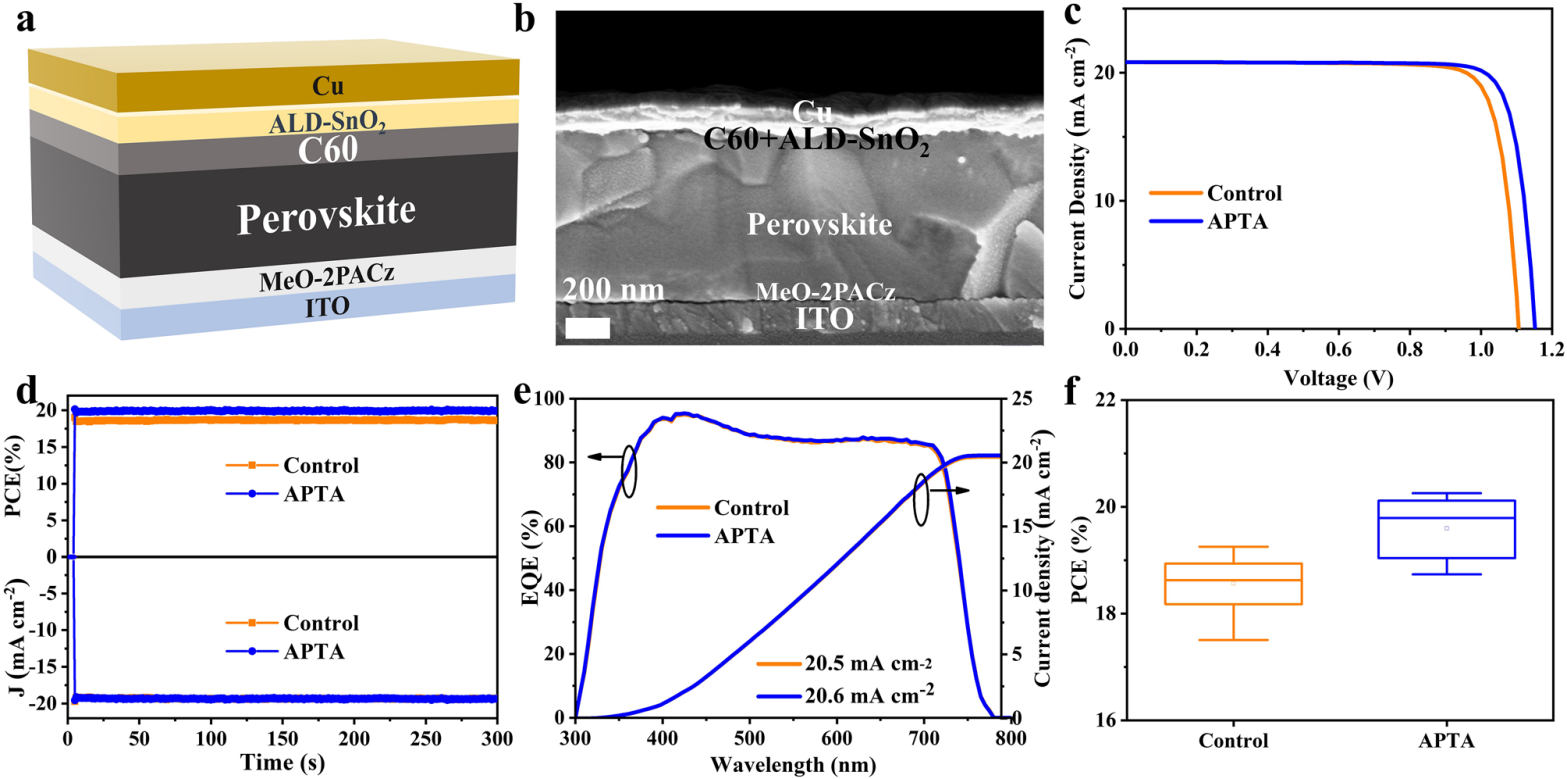
**a** Device structure of the inverted WBG PSC. **b** Cross-sectional SEM image of a WBG PSC. **c** *J–V* curves of WBG devices without and with APTA incorporation. **d** Steady-state power output and current densities of the devices measured under constant bias voltages. **e** EQE and integrated *J*_SC_ of the corresponding devices. f Statistical data of the PCE for WBG-based devices

Then, we fabricated the inverted WBG PSCs with the structure of ITO/[2-(3,6-dimethoxy-9H-carbazol-9-yl) ethyl] phosphonic acid (MeO-2PACz)/perovskite/C_60_/tin oxide (SnO_2_)/Cu, as shown in Fig. 3a. The corresponding cross-sectional SEM image of PSC is displayed in Figs. 3b and S3. It can be seen that the cross-sectional morphology of the APTA-assisted perovskite is better. C_60_ and MeO-2PACz are used as electron and hole transport layers, respectively. In addition, a stable and dense SnO_2_ upon atomic layer deposition (ALD) is used as a buffer layer to prevent oxygen and moisture from corroding perovskite film. The preparation process of PSCs devices is described in detail in the experimental part of supporting information. Figure S4 shows a statistical diagram of the device performance, which indicates that the optimal concentration of APTA is 1 mg mL^−1^. Figure 3c shows current–density versus voltage (*J*-*V*) curves of the hero PSCs without and with APTA in a reverse scan and the corresponding photovoltaic parameters are summarized in Table 1. The control device delivers a PCE of 19.21% with an *V*_OC_ of 1.112 V, short-circuit current density (*J*_SC_) of 20.8 mA cm^−2^, and fill factor (FF) of 83.2%. The low *V*_OC_ originates from the severe nonradiative recombination caused by traps in the WBG perovskite films. By contrast, the PSC with APTA exhibits an improved PCE of 20.35% with a *V*_OC_ of 1.153 V, *J*_SC_ of 20.9 mA cm^−2^, and FF of 84.5%. Both *V*_OC_ and FF contribute to the ultimate PCE enhancement, which may be attributed to the additive's effective passivation of defects. The open-circuit voltage losses for WBG devices with APTA decreases from 0.558 to 0.517 V. The *J*–*V* curves measured in both reverse and forward directions of the champion PSCs are shown in Fig. S5. Noted that the hysteresis is significantly reduced with the addition of APTA. The stabilized current density near the maximum power point of the PSCs with and without APTA and the corresponding power output are shown in Fig. 3d. It is evident that the stabilized power outputs do not decay at all within 5 min illumination for both devices. The steady-state efficiencies of the two devices are 19.01% and 20.10%, respectively. External quantum efficiency (EQE) spectra and the corresponding integrated *J*_SC_ of control and APTA devices are shown in Fig. 3e. It can be seen that the integrated *J*_SC_ both match the *J*_SC_ values from the *J*–*V* curves. Figure S6 provides the first-order derivatives of EQE spectra. The minimum value of the EQE differential spectrum both locate at 742 nm, being consistent with the bandgap value of 1.67 eV obtained from PL. The nearly equal integral *J*_SC_ indicates that APTA neither enhances absorption nor expands band gap of perovskite. Furthermore, 20 independent devices under the same manufacturing conditions were fabricated, and the statistical distribution of photovoltaic parameters is exhibited in Fig. 3f and Fig. S7. The results reveals that the PCE varied with *V*_OC_ and FF, showing similar trends. As shown in Table S2, the average PCE of APTA-combined PSCs is 20.12 ± 0.33%. However, PSCs in the control group only achieved an average of 19.02 ± 0.32% PCE.

**Table 1:** Summarized photovoltaic parameters obtained from the *J − V* curves in reverse scans

Sample	*V*_OC_ (V)	*J*_SC_ (mA cm^−2^)	FF (%)	PCE (%)
Control	1.112	20.8	83.2	19.21
APTA	1.153	20.9	84.5	20.35

### Energy-Level Alignment and Defect Analysis

**Fig. 4:**
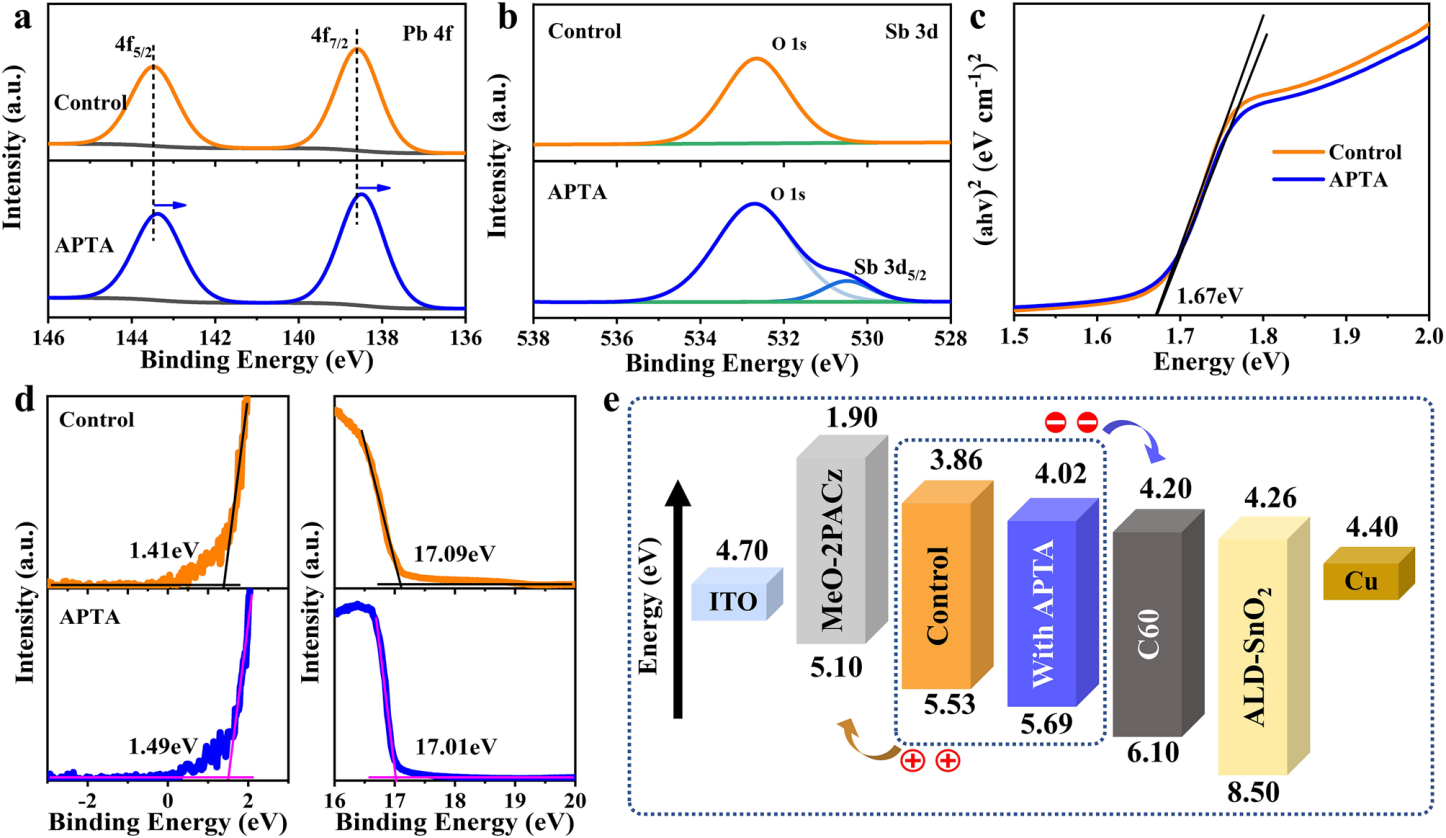
**a** Pb 4*f* and **b** Sb 3*d* XPS spectra for perovskite films without and with APTA. **c **Tauc plot and the corresponding optical bandgaps of perovskite films. **d** UPS spectra in the secondary electron cut-off and Fermi energy cut-off regions of the perovskite films. **e** Energy-level diagram for the inverted PSC device structure of ITO/MeO-2PACz/perovskite/C_60_/ALD-SnO_2_/Cu

To explore the underneath mechanism of APTA on perovskite formation, X-ray photoelectron spectroscopy (XPS) was conducted. Figure 4a shows the Pb 4*f* spectra for perovskite films with and without APTA. The peaks located at 143.3 and 138.4 eV are assigned to Pb 4f_5/2_ and 4*f*_7/2_ in the control film, respectively. Upon the addition of APTA, these two peaks shift to lower binding energies, suggesting an interaction between the APTA molecule and perovskite. That is probably due to the coordination of the carboxyl group in APTA with Pb, which decreases the binding energy of lead. The XPS spectra of other feature elements of perovskite are shown in Fig. S8. All the I 3*d* and Br 3*d* peaks slightly shift to higher binding energies, indicating the successful incorporation of K^+^ cation and the change of chemical environment caused by APTA [39]. In Sb 3*d* XPS spectra, as shown in Fig. 4b, the peak at ~ 530.6 eV in the perovskite film with APTA belongs to Sb 3*d*_5/2_, which is absent in the control, indicating of the presence of Sb from APTA. The strong peaks at around 532.7 eV are associated with O 1*s*, which may originate from oxygen or moisture in the air absorbed by the perovskite film during testing [40].

To evaluate the impact of APTA on energy band of perovskites, we then compared the *E*_g_ values of the two perovskite films, estimated by Ultraviolet visible (UV–vis) absorption spectra as shown in Fig. S9. Tauc plot and the corresponding optical bandgaps of perovskite films are shown in Fig. 4c. It can be seen that the influence of APTA on the absorption intensity and *E*_g_ of perovskite is almost invisible. Both perovskites own a bandgap of 1.67 eV, which is consistent with the PL and EQE spectra. Then, the effect of APTA on the electronic structure of perovskite thin films was studied using ultraviolet photoelectron spectroscopy (UPS), as shown in Fig. S10. The onset-point of valence band maximum (VBM) (*E*_onset_) and the secondary electron cutoff energy boundary (*E*_cutoff_) for both perovskite films are displayed in Fig. 4d. *E*_VBM_ can be calculated by the equation of *E*_VBM_ = 21.21 − *E*_cutoff_ + *E*_onset_. Finally, according to the *E*_g_ obtained above, we can calculate the conduction band minimum (ECBM) through the equation *E*_CBM_ = *E*_VBM_ – *E*_g_ [40, 41]. All of these parameters are summarized in Table S3. Although APTA does not change the band gap of perovskites, it can alter the band structure due to the cooperation of antimony. Compared with the control film, the Fermi level (*E*_F_) of the perovskite film with APTA is closer to the *E*_CBM_, which means that the film is closer to the n-type and can better match with the electron transport layer, resulting in higher *V*_OC_ and FF. The schematic diagram of the optimized energy band structure [17, 39, 42] for the whole PSC is shown in Fig. 4e.

**Fig. 5:**
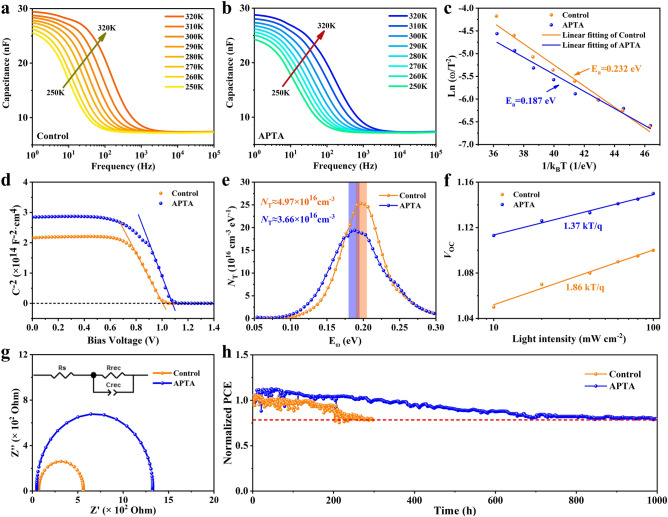
Admittance spectra of WBG PSCs **a** without and **b** with APTA incorporation. **c** The Arrhenius plots, **d** the Mott–Schottky plots, and **e** the trap state density at room temperature. **f** *V*_OC_ as a function of light intensities. **g** Nyquist plots of PSCs without and with APTA incorporation. **h** Continuous maximum power point (MPP) tracking for 1,000 h of PSCs without and with APTA under constant simulated solar illumination (100 mW cm^−2^) in nitrogen atmosphere

To further elucidate the effects of APTA on the trap distribution and energy, we carried out thermal admittance spectroscopy (TAS) measurements. For a semiconductor, the defect activation energy (*E*_a_) approximates the distance between the defect state energy level and the valence band [35, 43]. Herein, TAS measurements for the devices (Fig. 5a) without and (Fig. 5b) with APTA are executed at gradient temperatures (250 to 320 K) in the dark from 5 to 10,000 Hz. Figure S11 displays the derivative image of the capacitance–frequency spectra. The relationship between characteristic transition angular frequency ω0 and *E*_a_ follows Eq. 2:

2 $$\omega_{0} = \beta T^{2} e^{{ - \frac{{E_{a} }}{{k_{B} T}}}}$$

where *β* is a temperature-independent parameter, *T* the temperature in Kelvin, and *k*_B_ the Boltzmann constant. The Arrhenius plots (Fig. 5c) derived from the characteristic transition angular frequency *ω*_0_ and fitted curves indicated that the Ea of the control and APTA devices are 0.232 and 0.187 eV, respectively.

The built-in potential (*V*_bi_) and the depletion width (W) in the perovskite layer can be estimated by the depletion approximation equation (Mott–Schottky):

3 $$\frac{{A^{2} }}{{C^{2} }} = \frac{{2\left( {V_{bi} - V} \right)}}{{q\varepsilon \varepsilon_{0} N}}$$

where *A* is the active area of devices, *C* the capacitance, *N* the doping density, *V* the voltage applied on the devices, *q* the electron charge, *ε* and *ε*_0_ the relative permittivity (here with reference to 24.4 [23, 25]) and vacuum permittivity, respectively. The linear region of the Mott–Schottky plots reflects the property of the depletion layer, where the slope reflects the doping density N in perovskites and the intersection with the bias voltage is *V*_bi_ [44–46]. As shown in Fig. 5d, we measured the Mott–Schottky curves of PSCs with bias voltages ranging 0 to 1.4 V under a frequency of 1000 Hz at room temperature. The *V*_bi_ of the PSCs without and with APTA-incorporated are 1.01 and 1.09 V, respectively. Obviously, the improvement of *V*_bi_ is mainly attributed to the reduction of non-radiative recombination after the incorporation of APTA, which leads to less internal and interfacial voltage loss. The depletion width *W* can be obtained by Eq. 4:

4 $$W = \sqrt {\frac{{2\varepsilon \varepsilon_{0} V_{bi} }}{qN}}$$

The *W* of the PSCs without and with APTA-incorporated calculated by the above formula are 72 and 76 nm, respectively. The energy distribution of the trap density of states in the perovskite film can be obtained from Eqs. 5 and 6:

5 $$N_{T} \left( {E_{\omega } } \right) = - \frac{{V_{bi} }}{{qWk_{B} T}}\frac{\omega dC}{{d\omega }}$$

6 $$E_{\omega } = k_{B} T\ln \left( {\frac{{\beta T^{2} }}{\omega }} \right)$$

As is displayed in Fig. 5e, the trap state level of the WBG perovskite absorber decreases from 0.21 to 0.18 eV upon the appearance of APTA. The integrated trap density reduces from 4.97 × 10^16^ to 3.66 × 10^16^ cm^−3^. This means the WBG perovskite with APTA owns reduced nonradiative recombination, which is consistent with the PL measurements.

In order to Fig. out the deeper reasons for the device performance improvement, the dependence of *J-V* characteristics on light intensity was performed to study the carrier recombination dynamics. As shown in Fig. 5f. *V*_OC_ increases at the incident light intensity Φ_ph_, following a logarithmic relationship and the ideal factors of PSCs can be calculated with Eq. 7:

7 $$V_{OC} = \frac{{nk_{B} T}}{q}\ln \left( {{\Phi }_{ph} } \right)$$

where n is the ideal factor and* q* is the elementary charge. Through linear fitting, the slopes of the devices without and with APTA are 1.86 (k_B_T/q) and 1.37 (k_B_T/q), respectively. An ideal factor closer to 1 for the APTA-incorporated PSC indicated that trap-assisted recombination is effectively inhibited because of the reduction of trap density, which is consistent with the previous experimental results.

In addition, electrochemical impedance spectra (EIS) measurements were carried out to study the charge transport behavior of PSCs, and Nyquist plots are displayed in Fig. 5g and the inset picture shows the fitted equivalent circuit diagram in Fig. 5g. The increase of bias expedites the accumulation of holes at the interface between perovskite and HTL, which increases the recombination of electrons and holes, making series resistance (*R*_s_) and the recombination resistance (*R*_rec_) inevitably dependent on the *V*_OC_. As listed in Table S4, the *R*_s_ was reduced from 70.1 to 49.8 Ω and *R*rec was increased from 491.2 to 1274.5 Ω after the addition of APTA, suggesting that the carrier recombination is effectively suppressed by APTA additive. Furthermore, apart from efficiency, stability is also a crucial indicator of future commercial use of PSCs, so we tested the stability differences under continuous illumination after the additive. Due to the additive effect, after 1,000 h of MPP tracking under one continuous standard solar irradiation (AM1.5, 100 mW cm^−2^) at ≈ 55 ºC temperature, APTA-base PSCs can maintain more than 80% of their initial efficiency, while the efficiency of the control group devices continues to decline, its T80 is only 500 h (Fig. 5f).

### All Perovskite Tandem Solar Cell

**Fig. 6:**
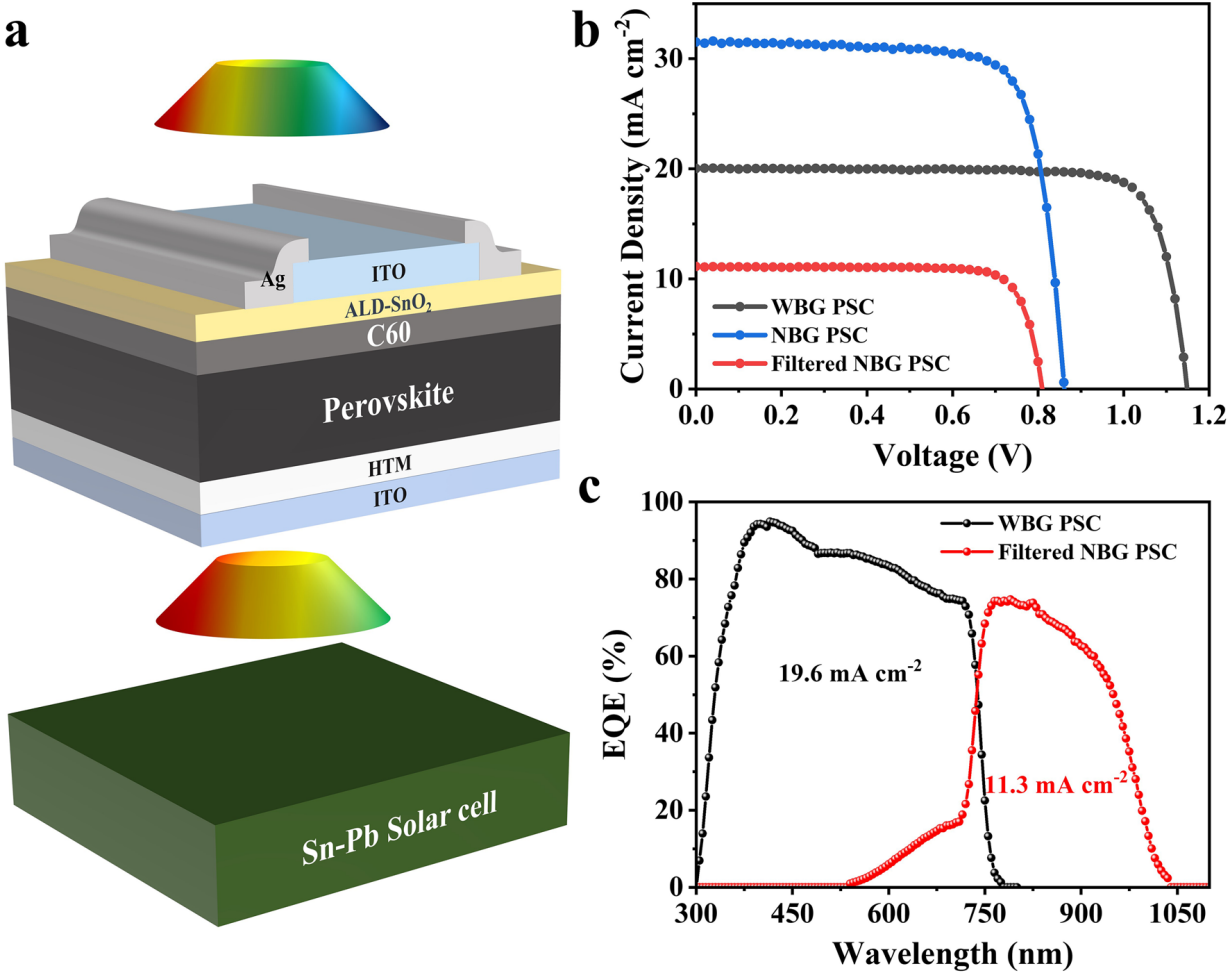
**a** Schematic device configuration of a 4-T perovskite/perovskite tandem cell. **b** *J*-*V* curves and **c** EQE spectra of a 4-T perovskite/perovskite tandem cell

We finally fabricated 4-T perovskite/perovskite tandem solar cell based on the WBG perovskite with APTA. Here, narrow-bandgap (NBG) Sn–Pb perovskites were fabricated by a spin-coating method, and the schematic diagram of device structure for Sn–Pb PSCs is displayed in Fig. S12. For the all-perovskite tandem devices, light is incident from the ITO/SnOx side of the semi-transparent WBG PSCs, and photons with higher energy are absorbed by the WBG top cells, while the photons with lower energy are absorbed by the NBG bottom cells. The corresponding schematic structure of tandem devices and the transmittance spectrum of semi-transparent WBG PSC are shown in Figs. 6a and S13, respectively. The *J-V* curves of semi-transparent WBG PSC, independent NBG PSC, and filtered NBG perovskite sub-cell are shown in Fig. 6b. The *J*-*V* curves of the semi-transparent WBG PSC with and without APTA are given in Fig. S14. Similar to the opaque devices, the APTA additive only improves the *V*_OC_ of semi-transparent cell with little effect on *J*_SC_. Compared with the device with metal electrodes, semi-transparent device yields lower efficiency, *J*_SC,_ and FF. The reduction of light absorption of perovskite caused by the transparent ITO top electrode accounts for the drop in *J*_SC_, which is consistent with the significant decline of the EQE spectra in the wavelength range from 530 to 710 nm as shown in Fig. 6c. The decrease in FF may be due to a sub-optimal contact interface. The semi-transparent WBG PSC demonstrated a PCE of 18.7%, while the NBG bottom cell showed a filtered PCE of 7.6%. Eventually, the combination of the top cell and the bottom cell resulted in an all-perovskite tandem cell with an efficiency of 26.3%. The corresponding photovoltage parameters of 4-T tandem cell are summarized in Table 2. This is one of the highest efficiencies reported for all-perovskite 4-T tandem solar cells as listed in Table S5. These superior results indicate that the incorporation of APTA improves both the stability and efficiency of WBG PSCs, further promoting the realization of efficient and stable all-perovskite tandem solar cells.

**Table 2:** Photovoltaic parameters of various types of solar cells

	*V*_OC_ (V)	*J*_SC_ (mA cm^−2^)	FF (%)	PCE (%)
Sn–Pb	0.859	31.5	76.7	20.8
Filtered Sn–Pb	0.817	11.5	80.9	7.6
WBG perovskite	1.146	19.9	81.6	18.7
4-T tandem				26.3

## Conclusions

In this work, we have demonstrated that the addition of APTA into perovskite precursor solution not only passivates the defects of perovskite but also inhibits phase segregation, which leads to the enhancement of carrier lifetime and stability of perovskite. Moreover, APTA also improves the energy-level alignment of WBG perovskites with charge transport layers, thereby effectively accelerating the extraction of carriers and suppressing the non-radiative interfacial recombination and hence the hysteresis of device. These effects synergistically lead to an efficient and stable WBG PSC with a PCE of 20.35%. Meanwhile, the improved device can maintain more than 80% of the initial efficiency after 1,000 h of MPP tracking under 1 sun illumination. In addition, we prepared a WBG semi-transparent cell with an efficiency of 18.7% with APTA additive and combined it with an NBG perovskite solar cell to obtain an all-perovskite 4T tandem solar cell with a PCE of 26.3%. Our results provide a facile method to modify the WBG perovskite for inverted planar PSCs, towards stable and efficient perovskite optoelectronic devices.

### Supplementary Information

Below is the link to the electronic supplementary material.

Supplementary file1 (PDF 1146 KB)
