# Supplementary material for: Antimony Potassium Tartrate Stabilizes Wide-Bandgap Perovskites for Inverted 4-T All-Perovskite Tandem Solar Cells with Efficiencies over 26%
Source: Nanomicro Lett. 2023 Apr 14;15:103. doi: 10.1007/s40820-023-01078-6 (PMC10105005; doi:10.1007/s40820-023-01078-6)
Supplement: Supplementary file 1 — Supplementary file1 (PDF 1146 KB) [file 40820_2023_1078_MOESM1_ESM.pdf]

Supporting Information for

Antimony Potassium Tartrate Stabilizes Wide-Bandgap Perovskites for Inverted 4T All-Perovskite Tandem Solar Cells with Efficiencies over 26%

Xuzhi Hu[1], Jiashuai Li[1], Chen Wang[1], Hongsen Cui[1], Yongjie Liu[1], Shun Zhou[1], Hongling Guan[1], Weijun Ke[1,*], Chen Tao[1,*] and Guojia Fang[1,*]

[1] Key Laboratory of Artificial Micro/Nano Structures of Ministry of Education, School of Physics and Technology, Wuhan University, Wuhan 430072, P. R. China

*Corresponding authors. E-mail: weijun.ke@whu.edu.cn (Weijun Ke); taochen635@whu.edu.cn (Chen Tao); gjfang@whu.edu.cn (Guojia Fang)

Supplementary Figures and Tables

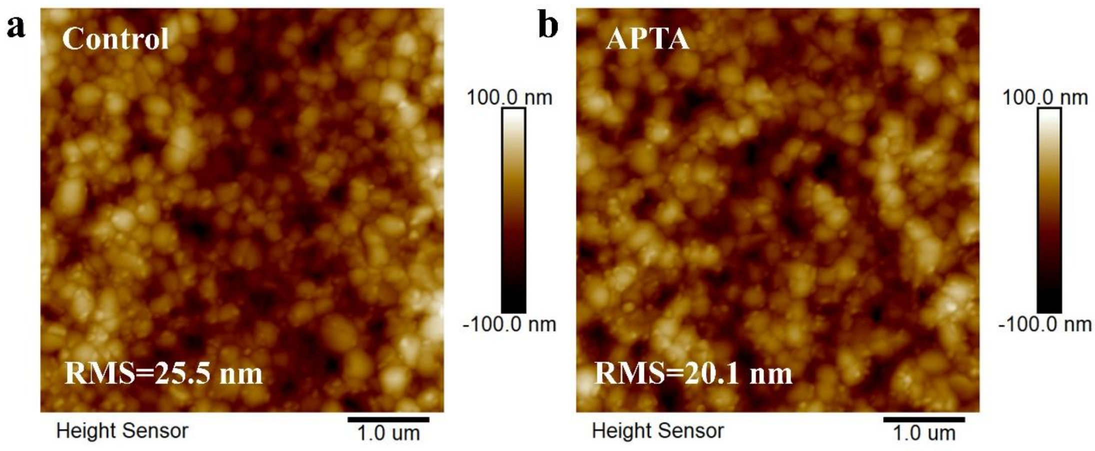

Fig. S1 AFM morphology and corresponding RMS roughness of perovskite **a** without and **b** with APTA additive

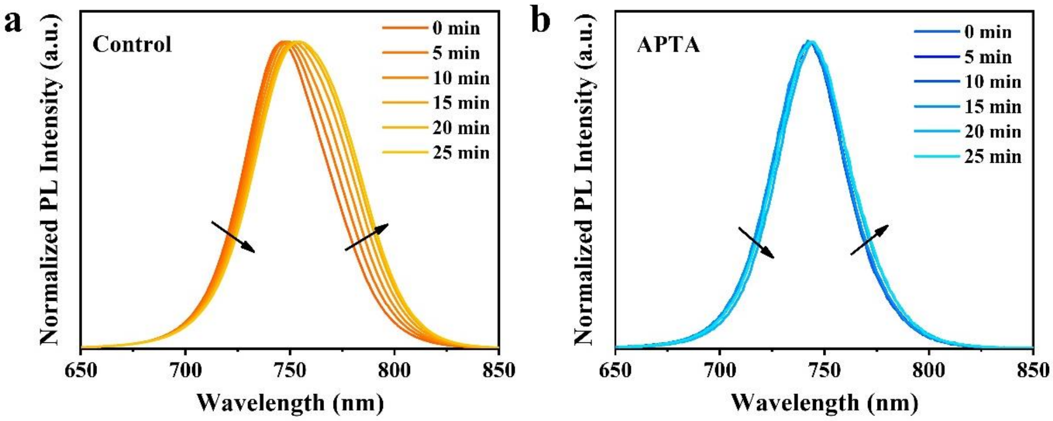

Fig. S2 PL peak changes of perovskite film **a** without and **b** with APTA under continuous laser irradiation

Table S1 Fitted Parameters of TRPL Spectra

samples	τ_1 (ns)	A_1	τ_2 (ns)	A_2	τ_{avg} (ns)
Control	30.23	33.88	254.62	66.12	241.75
APTA	65.14	23.20	852.44	76.80	834.46

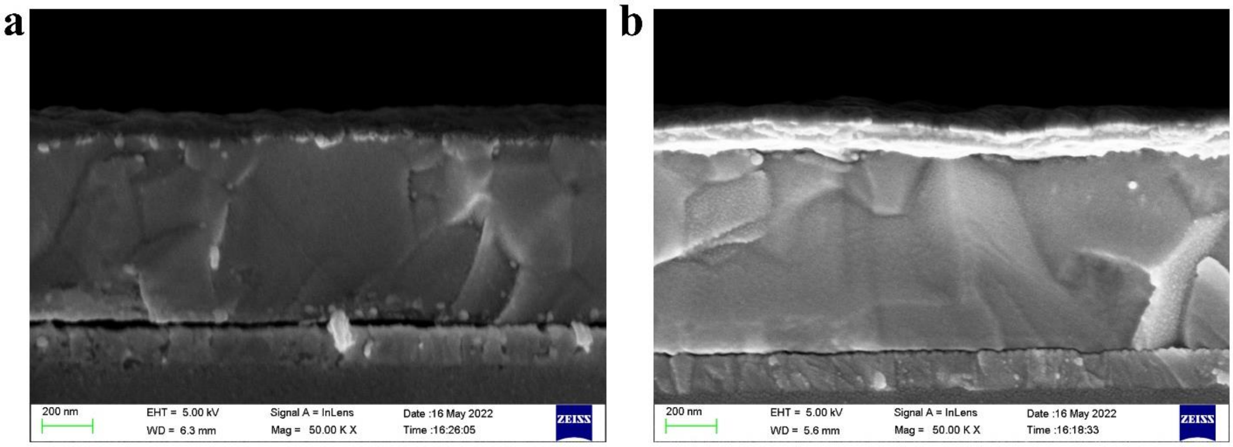

Fig. S3 Cross-sectional SEM image of PSC **a** without and **b** with APTA additive

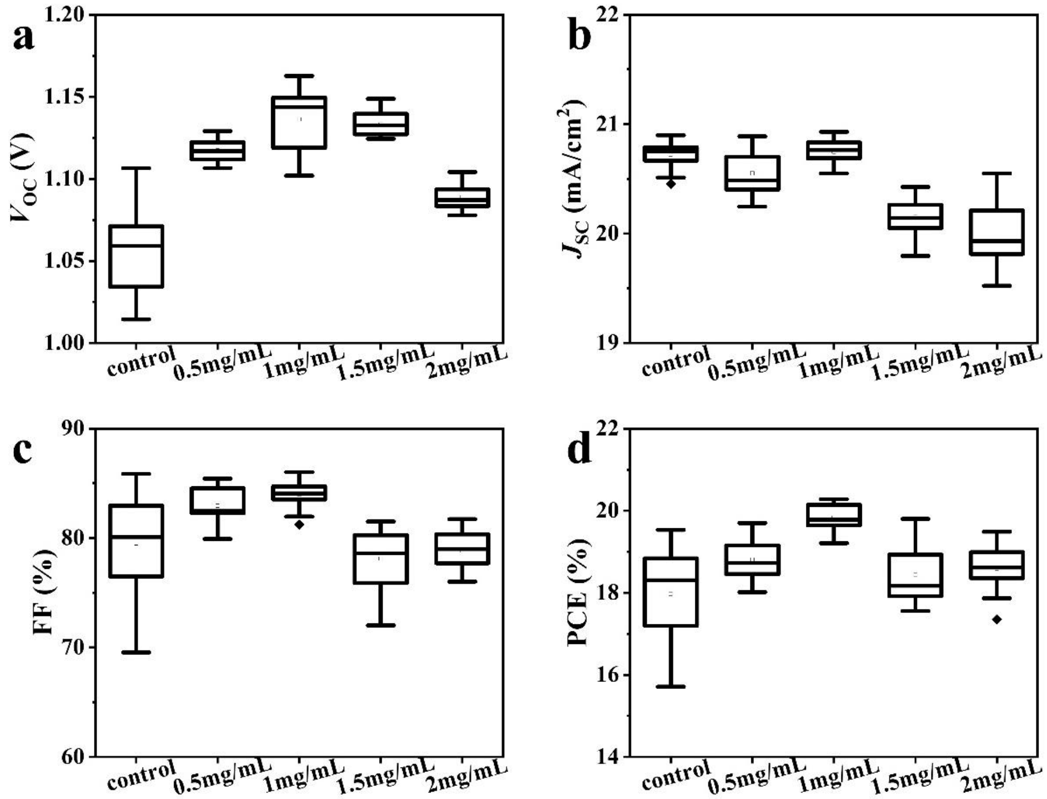

Fig. S4 The statistic performance parameters of **a** V_{OC}, **b** J_{SC}, **c** FF and **d** PCE for inverted WBG PSCs with different concentration of APTA

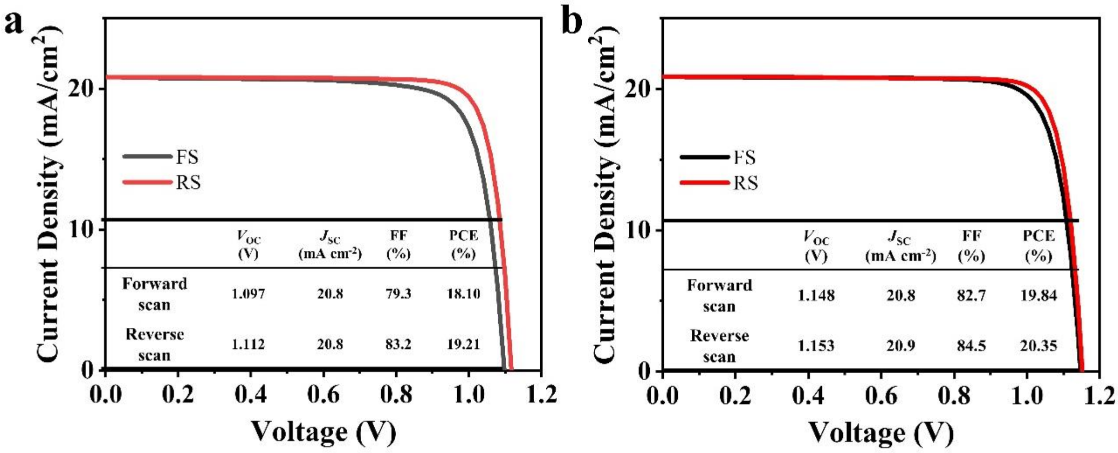

Fig. S5 *J–V* curves of the champion PSCs prepared **a** without or **b** with APTA measured in both reverse and forward directions, respectively. The photovoltaic parameters of the devices are both shown in each picture

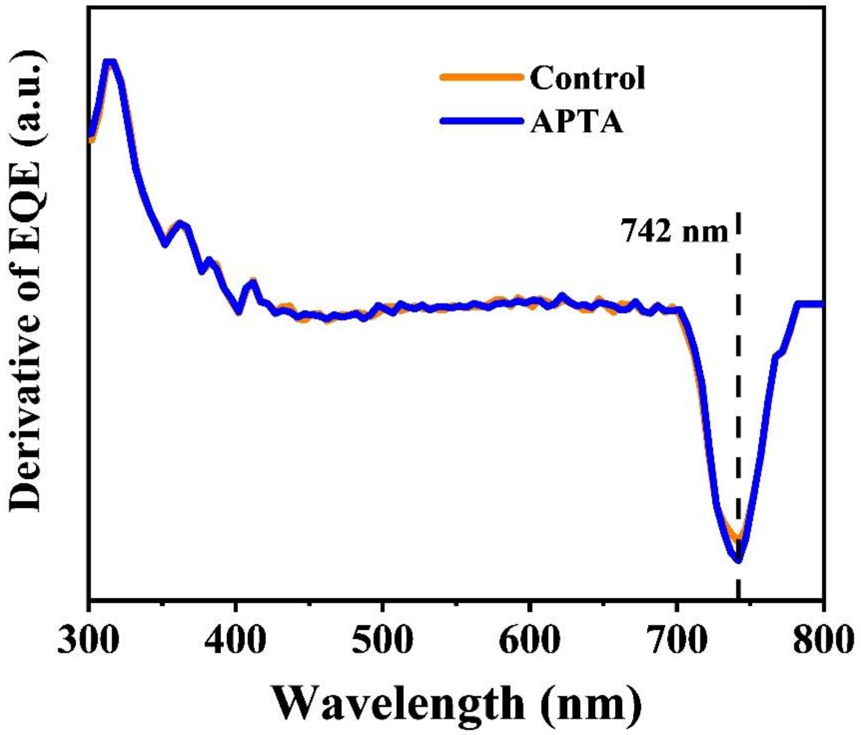

Fig. S6 The first derivative of the EQE curve

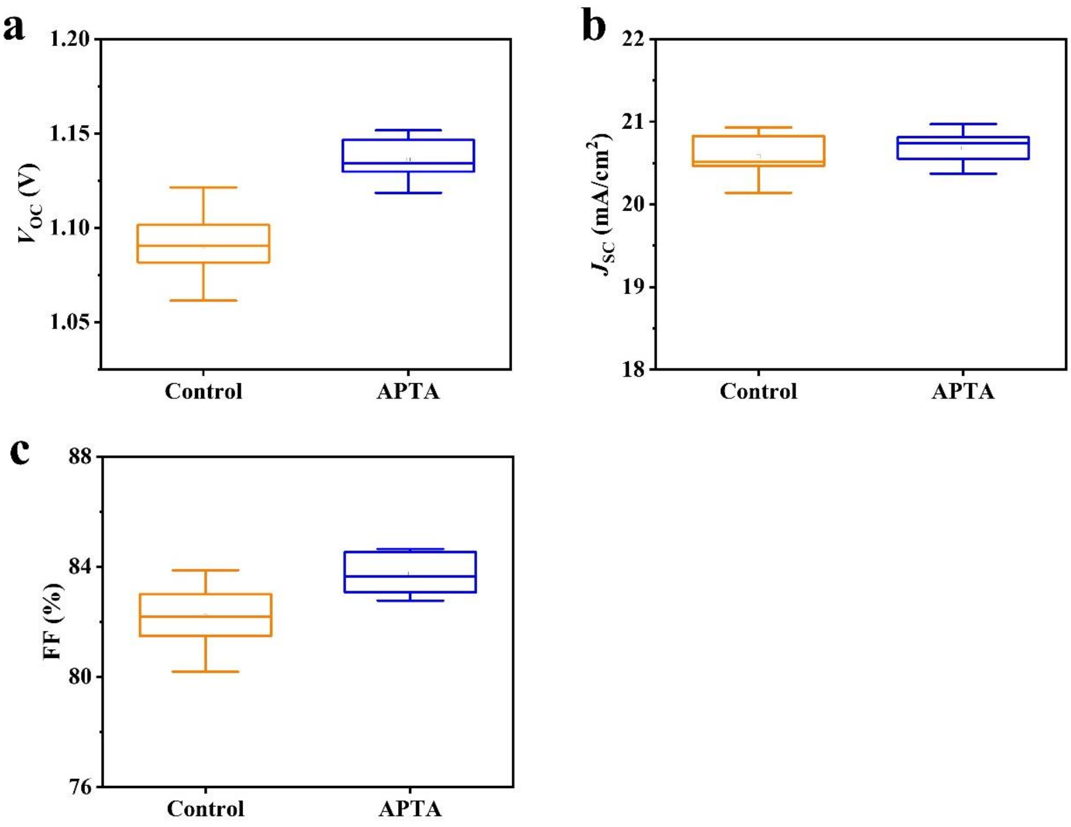

Fig. S7 Statistical data of **a** V_{OC}, **b** J_{SC} and **c** FF for WBG devices

Table S2 Standard deviations of the photovoltaic parameters of the WBG devices

sample	V_{OC} (V)	J_{SC} (mA cm^{-2})	FF (%)	PCE (%)
control	1.09±0.034	20.7±0.16	82.4±0.5	19.02±0.32
APTA	1.13±0.023	20.8±0.15	84.3±0.3	20.12±0.33

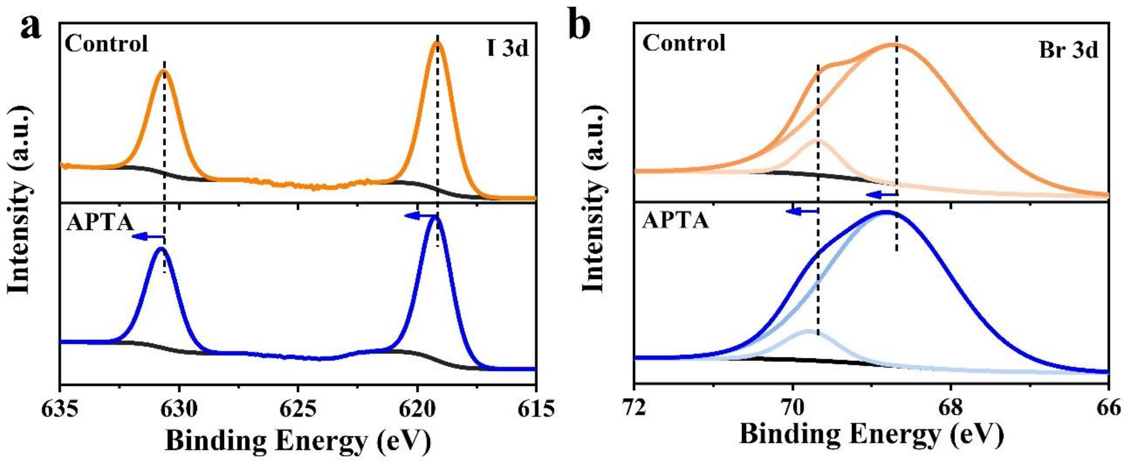

Fig. S8 a I 3d and **b** Br 3d XPS spectra for perovskite film w/o and with APTA

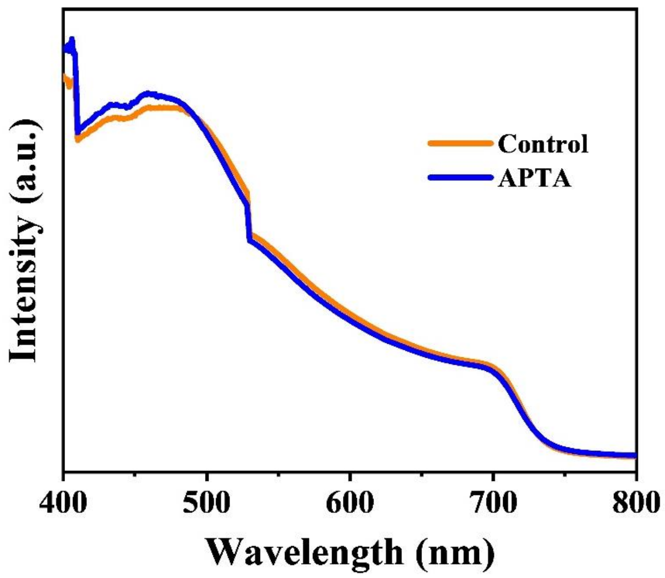

Fig. S9 UV–vis absorbance spectra of perovskite films w/o and with APTA

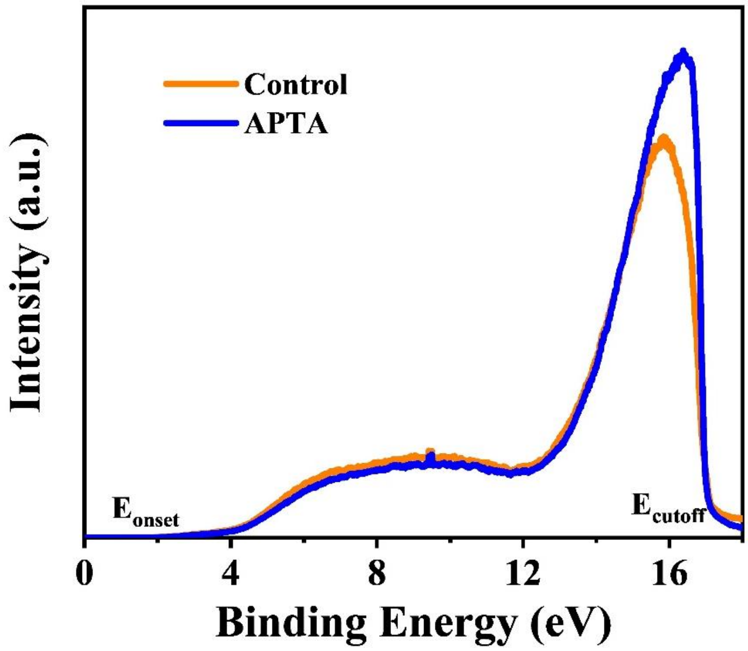

Fig. S10 UPS spectra of perovskite films w/o or with APTA

Table S3 Energy level parameters of different perovskites

samples	E_g (eV)	E_{onset} (eV)	E_{cutoff} (eV)	E_{VBM} (eV)	E_{CBM} (eV)	E_F (eV)
Control	1.67	1.41	17.09	5.53	3.86	4.12
APTA	1.67	1.49	17.01	5.69	4.02	4.20

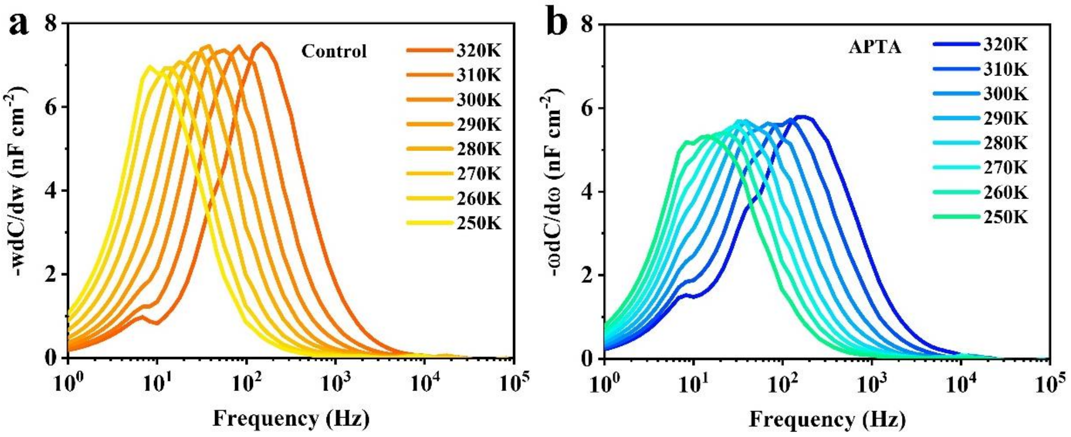

Fig. S11 The derivative of capacitance spectra of WBG devices **a** without and **b** with APTA incorporation

Table S4 EIS Parameters for the PSCs based on different perovskite

samples	Rs(Ω)	Rrec(Ω)
Control	70.1	491.2
APTA	49.8	1274.5

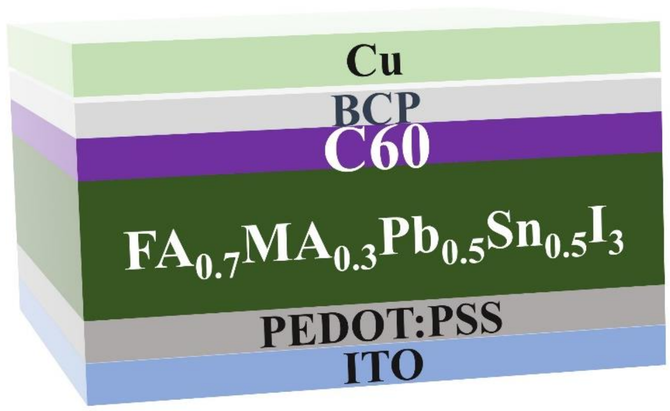

Fig. S12 Schematic device configuration of Sn-Pb NBG perovskite solar cell

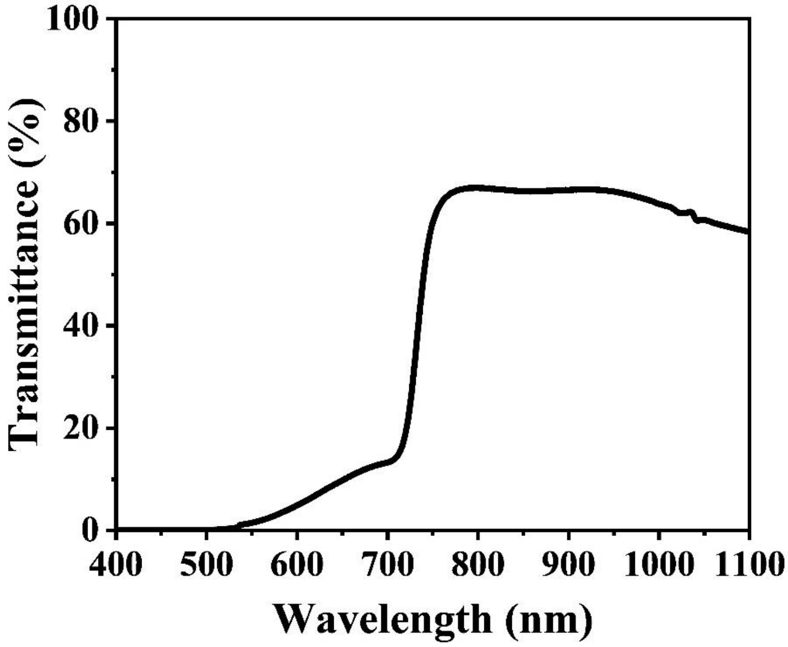

Fig. S13 Transmittance spectrum of semi-transparent perovskite top cell

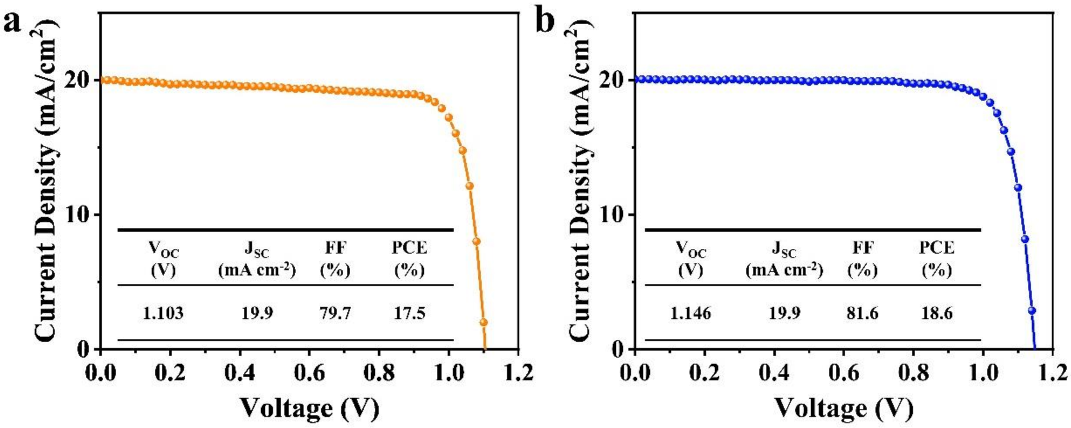

Fig. S14 J-V curves of the semi-transparent WBG PSC **a** without and **b** with APTA

Table S5 Summary of all-perovskite 4-T tandem solar cells

year	subcells	Device structure	PCE of 4-T tandem solar cells (%)	REFs
2016	top	ITO/NiO_x/$MAPbI_3$/PCBM/C60/ITO	19.08	[S1]
	bottom	ITO/PEDOT:PSS/$MA_{0.5}FA_{0.5}Sn_{0.25}Pb_{0.75}I_3$/PCBM/C60/Ag		
2016	top	ITO/NiO_x/$FA_{0.83}Cs_{0.17}Pb_{I0.83}Br_{0.17}$/PCBM/$SnO_2$/ZTO/ITO	20.1	[S2]
	bottom	ITO/PEDOT:PSS/$FA_{0.75}Cs_{0.25}Sn_{0.5}Pb_{0.5}I_3$/PCBM/BCP/Ag		
2017	top	FTO/SnO_2/C60-SAM/$MA_{0.7}FA_{0.3}PbI_3$/spiro-OMeTAD/MoO_x/Au/MoO_x	21.2	[S3]
	bottom	ITO/PEDOT:PSS/$(FASnI_3)_{0.6}(MAPbI_3)_{0.4}$/PCBM/BCP/Ag		
2017	top	ITO/NiOx/$MA_{0.9}Cs_{0.1}Pb(I_{0.6}Br_{0.4})_3$/C60/ C60/ITO	16.7	[S4]
	bottom	ITO/PEDOT:PSS/$MASn_{0.5}Pb_{0.5}I_3$/$IC_{60}BA$/ C60/Ag		
2018	top	FTO/SnO_2/C_{60}-SAM/$FA_{0.8}Cs_{0.2}Pb(I_{0.7}Br_{0.3})_3$/spiro-OMeTAD/MoOx/ITO	23.1	[S5]
	bottom	ITO/PEDOT:PSS/$(FASnI_3)_{0.6}(MAPbI_3)_{0.4}$/PCBM/BCP/Ag		
2020	top	ITO/SnO_2/$Cs_{0.1}(MA_{0.17}FA_{0.83})_{0.9}Pb(I_{0.83}Br_{0.17})_3$/spiro-OMeTAD/$MoO_x$/ITO	23.0	[S6]
	bottom	ITO/PEDOT:PSS/FA0.8MA0.2Sn0.5Pb0.5I3/PCBM/C60/BCP/Ag.		
2022	top	ITO/MeO-2PACz/$FA_{0.8}Cs_{0.2}PbI_{0.8}Br_{0.2}$/$C_{60}$/$SnO_2$/ITO	26.0	[S7]
	bottom	ITO/PEDOT:PSS/$FA_{0.7}MA_{0.3}Sn_{0.5}Pb_{0.5}I_3$/$C_{60}$/BCP/Ag.		
2023	top	ITO/MeO-2PACz/$FA_{0.75}Cs_{0.25}PbI_{0.8}Br_{0.2}$/$C_{60}$/$SnO_2$/ITO	26.3	This work
	bottom	ITO/PEDOT:PSS/$FA_{0.7}MA_{0.3}Sn_{0.5}Pb_{0.5}I_3$/C60/BCP/Ag.		

Supplementary References

[S1] Z. Yang, A. Rajagopal, C. Chueh, S. B. Jo, B. Liu et al., Stable Low-bandgap Pb-Sn binary perovskites for tandem solar cells. Adv. Mater. **28**, 8990-8997 (2016). https://doi.org/10.1002/adma.201602696

[S2] G. E. Eperon, T. Leijtens, K.A. Bush, R. Prasanna, T. Green et al., Perovskite-perovskite tandem photovoltaics with optimized band gaps. Science **354**, 861 (2016). https://doi.org/10.1126/science.aaf9717

[S3] D. Zhao, Y. Yu, C. Wang, W. Liao, N. Shrestha et al., Low-bandgap mixed tin-lead iodide perovskite absorbers with long carrier lifetimes for all-perovskite tandem solar cells. Nat. Energy **2**, 17018 (2017). https://doi.org/10.1038/nenergy.2017.18

[S4] A. Rajagopal, Z. Yang, S. B. Jo, I. L. Braly, P. W. Liang et al., Highly efficient perovskite-perovskite tandem solar cells reaching 80% of the theoretical limit in photovoltage. Adv. Mater. **29**, 1702140 (2017). https://doi.org/10.1002/adma.201702140

[S5] D. Zhao, C. Wang, Z. Song, Y. Yu, C. Chen et al., Four-terminal all-perovskite tandem solar cells achieving power conversion efficiencies exceeding 23%. ACS Energy Lett. **3**, 305 (2018). https://doi.org/10.1021/acsenergylett.7b01287

[S6] B. A. Nejand, I. M. Hossain, M. Jakoby, S. Moghadamzadeh, T. Abziehe et al., Vacuum-assisted growth of low-bandgap thin films ($FA_{0.8}MA_{0.2}Sn_{0.5}Pb_{0.5}I_3$) for all-perovskite tandem solar cells. Adv. Energy Mater. **10**, 1902583 (2020). https://doi.org/10.1002/aenm.201902583

[S7] W. Zhang, L. Huang, W. Zheng, S. Zhou, X. Hu, J. Zhou, J.Li, J, Liang, W. Ke, G. Fang. Revealing key factors of efficient narrow-bandgap mixed lead-tin perovskite solar cells via numerical simulations and experiments. Nano Energy **96**, 107078 (2022). https://doi.org/10.1016/j.nanoen.2022.107078
